# Functions of *Drosophila* Toll/NF-κB signaling in imaginal tissue homeostasis and cancer

**DOI:** 10.3389/fcell.2025.1559753

**Published:** 2025-03-12

**Authors:** Fabienne Brutscher, Konrad Basler

**Affiliations:** Department of Molecular Life Sciences, University of Zurich, Zurich, Switzerland

**Keywords:** *Drosophila*, innate immune signaling, Toll/NF-κB pathway, cell competition, tissue homeostasis, cell death, cancer

## Abstract

The Toll/NF-κB pathway plays a central role in patterning the *Drosophila* embryo and in orchestrating the innate immune response against microbial infections. Both discoveries were associated with a Nobel Prize award and led to the recognition of the Toll-like receptor pathway in mammals, which has significant implications for diseases. Recent discoveries have revealed that the Toll/NF-κB pathway also maintains epithelial homeostasis of imaginal tissues during development: local Toll/NF-κB signaling activity monitors internal cellular fitness, and precancerous mutant cells can trigger systemic Toll/NF-κB pathway activation. However, this signaling can be exploited in diseases like cancer, in which Toll/NF-κB signaling is often co-opted or subverted. Various models have been proposed to explain how Toll/NF-κB signaling contributes to different types of cancer. Here we provide an overview of the functions of Toll/NF-κB signaling in imaginal tissue homeostasis with a focus on their misuse in pathological contexts, particularly their significance for tumor formation.

## 1 The Toll/NF-κB pathway

The Toll/NF-κB pathway is a central player in the *Drosophila* embryonic pattern formation (Nüsslein-Volhard and Wieschaus, 1980) and in the innate immune response against microbial infections (Lemaitre et al., 1996); both discoveries were associated with a Nobel Prize award and led to the recognition of the evolutionarily conserved Toll-like receptor (TLR) pathway in mammals and its pathological significance.

In *Drosophila*, there are nine Toll-related receptors (TRRs). The roles of Toll/NF-κB signaling in embryonic patterning and innate immunity are mainly based on the originally identified Toll receptor, Toll-1 (Tl-1) (Nüsslein-Volhard, 2022; Valanne et al., 2022). The eight additional TRRs are suggested to activate the Toll signaling cascade mostly in non-immune contexts during development and adulthood. For a comprehensive review see Anthoney et al. (2018). The homologous mammalian TLRs, of which there are ten in humans, recognize diverse microbial products thereby collectively regulating innate immunity against a broad spectrum of pathogens (Gay and Gangloff, 2007). In contrast to the *Drosophila* TRRs, mammalian TLRs are not considered to be involved in early mammalian development.

*Drosophila* TRRs bind an activated form of Spätzle (Spz), whereas mammalian TLRs directly recognize proteins, such as those from pathogens or released by injuries (Gay and Gangloff, 2007). Spz 1-6, which are members of the neurotrophin-protein family, are produced as pro-proteins. The pro-proteins are then cleaved through a serine-protease cascade in response to developmental cues during embryogenesis or extracellular stimuli, such as Gram-positive bacterial or fungal infection (Stathopoulos and Levine, 2002; Morisato and Anderson, 1994; Weber et al., 2003).

Both, *Drosophila* TRR and mammalian TLR activation trigger the canonical Toll/NF-κB signaling pathway (for simplicity hereafter referred to as the Toll pathway). Via the evolutionary conserved Toll intracellular (TIR) domain, the TRRs and TLRs recruit Myd88, which in turn activates the protein kinase Pelle or IRAK (in mammals). In *Drosophila* Pelle then phosphorylates the *Drosophila* l-κB factor Cactus, targeting it for proteasomal degradation (Horng and Medzhitov, 2001; Wu and Anderson, 1998; Sun et al., 2002). Consequently, the NF-κB transcription factors Dorsal (Dl) and Dif are no longer retained in the cytoplasm but can translocate to the nucleus and regulate the expression of distinct target genes (Bulet et al., 1999). Similarly, in mammals, the canonical signaling cascade downstream of TLR activation ultimately results in the nuclear translocation of NF-κB transcription factors to activate transcription (Kawai and Akira, 2007).

Pioneering experiments in *Drosophila* on the role of innate immune signaling in response to mutant cells during imaginal epithelial development revealed new Toll pathway functions: Internal cellular fitness is monitored by healthy cells that locally activate Toll signaling in adjacent mutant cells (Meyer et al., 2014). Additionally, systemic Toll pathway activation triggered by polarity-deficient epithelia maintains tissue homeostasis to prevent oncogenic transformation of imaginal tissues during larval development (Parisi et al., 2014). These findings paved the way for studies on Toll signaling in cancer, revealing its dual functionality. For example, activating the Toll pathway in polarity-deficient epithelial cancer clones mutant for *scribble* no longer protects tissue homeostasis, but instead promotes tumor growth (Katsukawa et al., 2018). Thus like in mammals (Perkins, 2004; Perkins and Gilmore, 2006), in *Drosophila* models, the consequences of Toll signaling in cancer are context-dependent and the underlying studies help to advance our understanding of the mammalian TLR pathway’s functionality.

Here we review the functions of Toll signaling in imaginal tissue homeostasis with a focus on their misuse in pathological contexts, specifically their contribution to tumor formation.

## 2 The functions of Toll/NF-κB signaling in imaginal tissue homeostasis

### 2.1 Regulation of cell competition

Cell competition describes the non-cell autonomous elimination of cells in a heterotypic setting. The eliminated cells, often referred to as “loser cells”, would otherwise persist if they were not intermingled with more competitive cells. Cell competition is crucial for monitoring cell fitness and maintaining tissue homeostasis (referred to as “canonical cell competition”) but can also be misused by precancerous cells to promote oncogenic clonal expansion (referred to as “super-competition”, see Section 3.1.2). For a comprehensive recent review on cell competition, see Nagata and Igaki (2024).

**FIGURE 1:**
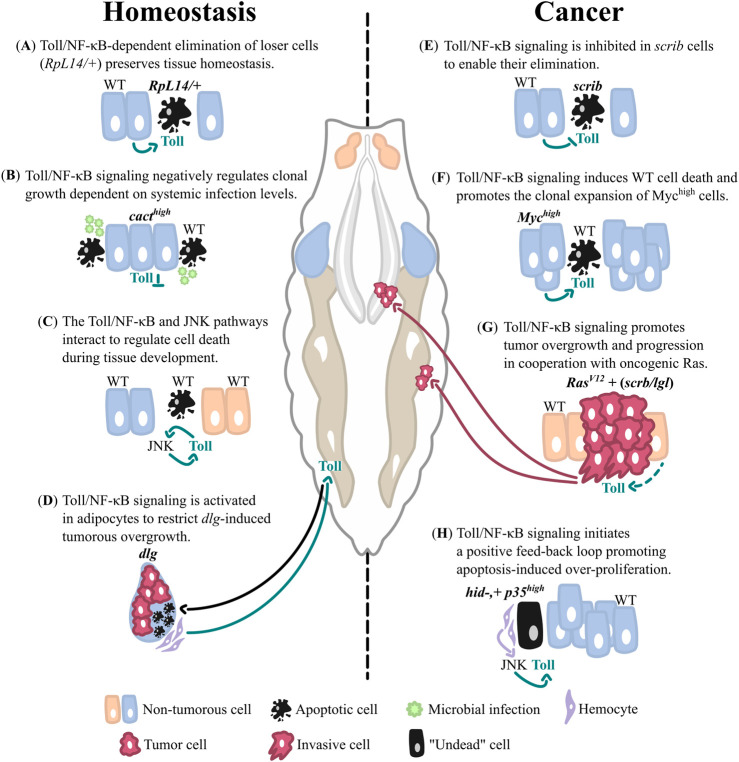
The functions of Toll/NF-κB signaling in imaginal tissue homeostasis and cancer. Schematic representations of the different contexts in which Toll signaling regulates tissue homeostasis and contributes to tumorous overgrowth in *Drosophila* imaginal tissues. The central scheme shows a *Drosophila* larva with the eye/antennal-(orange) and wing (blue) imaginal discs, the salivary glands (grey) and adipose tissue (brown). **(A–D)** The Toll pathway is critical for maintaining imaginal tissue homeostasis and protecting from oncogenic overgrowth: **(A)** Toll signaling is activated in *RpL14/+* mutant cells to regulate loser cell elimination. **(B)** Toll pathway inhibition confers cells with a growth advantage over surrounding WT cells. **(C)** The Toll and JNK pathways interact to regulate developmental cell death. **(D)** The systemic activation of Toll signaling in adipocytes protects from tumorous overgrowth in response to tissue-wide loss of apicobasal polarity. **(E–H)** Toll signaling outputs are co-opted to promote cancer development **(E)** Toll signaling needs to be inhibited to enable the elimination of polarity-deficient cells and prevent their tumorous overgrowth. **(F)** Cells with an activating mutation in Myc induce Toll-dependent cell death in neighboring WT cells to promote their oncogenic expansion. **(G)** Toll signaling promotes tumor growth and progression in cooperation with oncogenic Ras. **(H)** Toll signaling is required in “undead” cells to initiate a feedback amplification loop and promote overgrowth of surrounding WT cells. Dashed lines represent uncertain connections, blunt arrows (┴) indicate inhibition while sharp arrows (→) indicate stimulation or cell movement to distant tissues (magenta arrow in **(G)**). Inhibition or stimulation of Toll signaling is shown by green arrows, stimulation of cell death in **(D)** is illustrated by a black arrow and stimulation of JNK signaling specifically by hemocytes is shown by a purple arrow in **(H)**. WT, wild type.

A classical paradigm of cell competition is the elimination of wing imaginal epithelial cells heterozygous for a mutation in a ribosomal protein gene (hereafter referred to as *RpL14/+*). The elimination requires local activation of Toll signaling in loser cells (Figure 1A) (Meyer et al., 2014). The signaling module downstream of TRR activation relies on the NF-κB transcription factors Dl and Dif to activate expression of the pro-apoptotic gene *reaper* (*rpr*) and induce apoptotic cell death (Meyer et al., 2014; Morata and Ballesteros-Arias, 2014). The mechanism of Toll pathway activation in *RpL14/+* cells is still unclear, but may involve Spz as a ligand for the TRRs, Tl-3, and Tl-9 (Meyer et al., 2014) (Table 1). Adding an additional layer of complexity, a follow-up study demonstrates that activation of Toll signaling in this context is not exclusively dependent on locally produced Spz, but also on the systemic level of infection (Germani et al., 2018). This study shows that in a heterotypic population of cells with different levels of Toll signaling activity, cells with higher intrinsic Toll signaling activity were competitively eliminated (Germani et al., 2018) (Figure 1B). Taken together, both studies (Meyer et al., 2014; Germani et al., 2018) provide compelling evidence for a link between innate immunity and cell competition, where local activation of Toll signaling protects imaginal tissue homeostasis by promoting the elimination of “loser” cells.

**TABLE 1:** TRR-Spz interactions and their function in imaginal tissue homeostasis and cancer.

TRR	FlyBase ID	Ligand	NF-κB	Target	Function	Reference
Tl-1	FBgn0262473	Spz-1	Rel	*hid*	Regulation of loser cell death in *Myc*-induced cell competition	Alpar et al. (2018)
		Spz-1–6	Dl, Dif	“Cell death”	Regulation of *egr*-induced cell death in the eye-antennal disc	Wu et al. (2015a)
		Spz-1	Dl	*hid, rpr*	Amplification of AiP-induced overgrowth	Shields et al. (2022)
Tl-2/18w	FBgn0287775	—	Rel	*hid*	Regulation of loser cell death in *Myc*-induced cell competition (suggested interaction with Tl-8)	Meyer et al. (2014)
Tl-3/MstProx	FBgn0015770	—	Rel	*hid*	Regulation of loser cell death in *Myc*-induced cell competition (suggested interaction with Tl-8)	Meyer et al. (2014)
		Spz-1	Dl, Dif	*rpr*	Regulation of loser cell death in *RpL14/+*-induced cell competition	Meyer et al. (2014)
Tl-4	FBgn0032095	—	—	—	—	—
Tl-5/Tehao	FBgn0026760	—	—	—	—	—
Tl-6	FBgn0036494	Spz-5	—	JNK	Guides organotropic metastasis in *Ras* ^*V12*^ *, lgl*-induced tumorigenesis	Mishra-Gorur et al. (2019)
		Spz-5	NF-κB-independent	Yki	Regulation of *scrib*-induced cell competition	Kong et al. (2022)
Tl-7	FBgn0034476	—	—	JNK-Yki EGFR	Support of Ras^V12^, *lgl*-dependent tumor growth and invasion	Ding et al. (2022)
Tl-8/Tollo	FBgn0029114	Spz-1	Rel	*hid*	Regulation of loser cell death in *Myc*-induced cell competition	Meyer et al. (2014)
Tl-9	FBgn0036978	—	Rel	*hid*	Regulation of loser cell death in Myc-induced cell competition (suggested interaction with Tl-8)	Meyer et al. (2014)
		Spz-1	Dl, Dif	*rpr*	Regulation of loser cell death in *RpL14*-induced cell competition	Meyer et al. (2014)
		—	—	—	Lateral interaction with Tl-1 in AiP-dependent overgrowth	Shields et al. (2022)

Interestingly, recent findings suggest that TLR signaling may also be involved in the induction of loser cell apoptosis during cell competition in human cells (Zheng et al., 2021). The role of Toll signaling in cell competition may thus be conserved.

### 2.2 Regulation of JNK-mediated developmental cell death

Robust development of imaginal epithelia occasionally requires that cells, i.e., excess or misplaced cells, are eliminated by cell death (Wolff and Ready, 1991; Raff, 1992). The evolutionarily conserved c-Jun N-terminal kinase (JNK) pathway is a fundamental regulator of cell death during development (Adachi-Yamada et al., 1999). In the context of cell competition, the contribution of the JNK pathway to Toll signaling-mediated apoptotic death of “loser” cells, is debated (Meyer et al., 2014; Katsukawa et al., 2018; Kodra et al., 2024). However, several studies have addressed the epistatic relationship between the Toll and JNK pathways in developmental cell death (Figure 1C) (Wu et al., 2015a; Li et al., 2020; Wu et al., 2015b).

Toll signaling is essential for *egr*-induced, JNK-dependent cell death in eye/antennal and wing imaginal epithelia (Wu et al., 2015a). The Toll pathway acts downstream of JNK-activated forkhead box transcription factor (FoxO) and was suggested to induce cell death independent of caspases (Wu et al., 2015a); the exact mechanism remains unclear. Elevated JNK signaling during development can directly induce Toll pathway activity, supposedly by regulating the expression of *spz-6* in the epithelial peripodial membrane (Table 1) (Wu et al., 2015a). Interestingly, another study showed that ectopic Toll pathway activation in the wing imaginal epithelium can induce JNK signaling, leading to caspase-dependent cell death through an increase in reactive oxygen species (ROS) (Li et al., 2020). Based on these two examples of crosstalk, it is tempting to speculate there may be a positive feedback connection between the Toll and JNK pathways. There may also be a negative feedback mechanism to limit JNK-induced cell death in response to high levels of Toll pathway activity: The Toll pathway kinase Pelle can inhibit JNK- and caspase-dependent cell death through physical interaction with FoxO (Wu et al., 2015b). This interaction is independent of the NF-κB factor Dl (Wu et al., 2015b).

Thus, although the link between the Toll and JNK signaling pathways in developmental cell death has been repeatedly demonstrated (Wu et al., 2015a; Li et al., 2020; Wu et al., 2015b), further studies are needed to explain how the two pathways interact and crosstalk to modulate cell death during imaginal epithelial development.

### 2.3 Protection against tumorous overgrowth

The presence of tumorigenic cells in imaginal epithelia presents an internal threat to tissue integrity and organismal survival. *Drosophila* has emerged as a model to study not only the effects of local but also systemic signaling changes in response to tumors (Bilder et al., 2021). Defective apicobasal polarity and chromosomal instability (CIN) are two tumorigenic stimuli that trigger systemic activation of the innate immune system (Pastor-Pareja et al., 2008; Cordero et al., 2010; García-López et al., 2021; Liu et al., 2015).

Three epithelial polarity proteins, Lethal giant larvae (Lgl), Scribble (Scrib) and Discs large (Dlg) are established tumor suppressors that, if lost homozygous throughout an entire imaginal epithelium, can cause uncontrolled proliferation and amorphous overgrowth (Gateff, 1978; Bilder et al., 2000; Brumby and Richardson, 2003). To counteract the overgrowth, *Drosophila* mounts a systemic immune response that involves amplification and recruitment of circulating hemocytes to the side of tumor formation (Pastor-Pareja et al., 2008). Hemocyte-derived Eiger (Egr)/Tumor necrosis factor α (TNFα) thereupon induces JNK-dependent tumor cell death (Cordero et al., 2010).

Importantly, tumor-associated hemocytes (TAHs) also secrete the TRR ligand Spz which activates Toll signaling in adipose tissue, further restricting tumor overgrowth in imaginal epithelia (Figure 1D) (Parisi et al., 2014). Long-range Toll signaling from adipocytes is thought to contribute to tumor cell death by stimulating the expression of antimicrobial peptides (AMPs), i.e., Defensin (Parvy et al., 2019). The AMPs target tumor cells through the phosphatidylserine (PS)-rich domains they expose (Parvy et al., 2019).

CIN, which is often associated with tumorigenesis in mammals and flies (Nowak et al., 2002; Dekanty et al., 2012; Barrio et al., 2023; Gerlach and Herranz, 2020), also induces local and systemic immune responses: ROS-triggered local activation of Toll signaling in CIN-affected cells is suggested to induce a systemic immune response and to mediate the effective JNK-mediated death of transformed epithelial cells (Liu et al., 2015).

The Toll pathway can also be hijacked and its output used to promote tumor growth and progression rather than tissue integrity.

## 3 Misuse of Toll/NF-κB signaling in cancer

Mammalian TLR signaling is often mis-regulated in pathological contexts, including in different human cancers (Gilmore et al., 2002). The below studies suggest that also in *Drosophila,* Toll signaling can drive overgrowth, tumor formation and progression towards malignancy.

### 3.1 Tumor cell expansion

#### 3.1.1 Polarity deficiency-induced cell competition

A tissue-wide homozygous loss-of-function mutation in tumor suppressors, such as in the polarity gene *dlg*, systemically activates Toll signaling in adipocytes to limit tumorous overgrowth, as discussed in Section 2.3 (Parisi et al., 2014). However, when polarity-deficient cells, such as those homozygous mutant for *scrib*, are surrounded by wild type (WT) tissue, they are competitively eliminated via localized activation of the JNK pathway (Brumby and Richardson, 2003; Agrawal et al., 1995; Igaki et al., 2009; Pagliarini and Xu, 2003; Snigdha et al., 2021). Additionally, interaction of Spz-5/Tl-6 at competitive cell boundaries is proposed to activate the Hippo pathway independent of NF-κB transcription factors, which further supports the elimination of *scrib* cells (Table 1) (Kong et al., 2022).

Interestingly, Toll signaling must be actively inhibited in *scrib* cells for their effective competitive elimination (Figure 1E) (Katsukawa et al., 2018). In contrast, ectopic activation of Toll signaling drives oncogenic expansion of these polarity-deficient cells (Katsukawa et al., 2018). Mechanistically, WT cells surrounding *scrib* mutants secrete the protease inhibitor Serpin 5 (Spn-5), which suppresses Spz-mediated Toll signaling in *scrib* cells. In the absence of Spn-5, *scrib* cells survive and overgrow due to Toll-mediated activation of the JNK and Yki signaling pathways.

Thus, immune signaling is suppressed in polarity deficient cells, as ectopic Toll pathway activation impairs their effective elimination and promotes tumor growth. Further experiments are necessary to investigate whether tumor-initiating alterations, when acquired clonally rather than through tissue-wide loss of apicobasal polarity, fail to trigger or remain unaffected by systemic Toll pathway activation.

#### 3.1.2 Myc-induced super-competition

During tumor development, an activating mutation in a growth-promoting gene, such as Myc (hereafter: oncogenic Myc or Myc) provides cells with a competitive growth advantage, also referred to as super-competition (Moreno and Basler, 2004; De La Cova et al., 2004).

Cells with elevated Myc expression are suggested to remain isolated from systemic tumor-suppressive immune signaling (Alpar et al., 2018), but they locally induce Toll signaling in surrounding WT “loser” cells (Figure 1F) (Meyer et al., 2014; Morata and Ballesteros-Arias, 2014). In the context of Myc super-competition, the Toll pathway is suggested to mediate loser cell death via activation of the pro-apoptotic genes *hid* and *rpr* (Meyer et al., 2014; Alpar et al., 2018). Crucial for the activation of the Toll pathway in WT loser cells is the increased expression of Spz and Spz-processing enzymes (SPE) in the Myc-expressing winner cells (Alpar et al., 2018). Activated Spz induces Toll signaling in WT loser cells mainly via TRRs Tl-1 and Tl-8 (Alpar et al., 2018). These receptors are downregulated in oncogenic Myc-expressing cells, pointing to a mechanism that ensures specificity in killing WT loser cells (Alpar et al., 2018).

In contrast to *RpL14/+*-induced cell competition, in the context of oncogenic Myc-induced super-competition, Toll-induced death of loser cells does not protect tissue integrity, but instead promotes the expansion of tumor cells.

It appears paradoxical that Toll signaling has opposite functions in two competitive contexts, both induced by the presence of tumor cells: Toll pathway activation induces the death of WT cells surrounding Myc cells but induces over-proliferation in polarity deficient cells. However, common to both contexts is that the activation of Toll signaling ultimately accelerates tumor growth by promoting the expansion of tumorous cells.

#### 3.1.3 Ras^V12^-transformed epithelia

The growth-controlling small GTPase, Ras, is frequently linked to mammalian cancer (Sanchez-Vega et al., 2018). Cells with an activating mutation in Ras (hereafter: oncogenic Ras or Ras^V12^) are resistant to apoptotic stimuli (Kurada and White, 1998; Bergmann et al., 1998). However, the acquisition of additional mutations are necessary to accelerate tumor growth and initiate the progression towards malignancy (Hanahan and Weinberg, 2000).

Recent findings in the eye/antennal imaginal epithelium show that ectopic Toll pathway activation is sufficient to promote overgrowth in tissues expressing oncogenic Ras (Figure 1G) (Brutscher et al., 2024; Dillard et al., 2024). It is suggested that Toll signaling mediates overgrowth by repressing differentiation and increasing proliferation in cells predisposed by oncogenic Ras (Brutscher et al., 2024). Brutscher et al. hypothesize that induction of caspase expression in response to Toll signaling activity, as seen in WT cells, may contribute to Ras^V12^-related tumorigenic overgrowth through apoptosis-induced proliferation (Brutscher et al., 2024).

Toll signaling is activated in malignant tumor cells with concomitant activation of oncogenic Ras and loss of apicobasal polarity. Even though the underlying mechanism is not yet fully elucidated, elevated expression of upstream components of the Toll pathway, such as PGRP-SA, are suggested to contribute to local Toll pathway activation in malignant tumor cells (Dillard et al., 2024). Interestingly, the level of Toll signaling appears heterogeneous in the imaginal epithelium (Dillard et al., 2024). The authors suggest it may account for spatially variable functions of Toll signaling within the tumor epithelium, which promote either overgrowth or tumor cell invasion through variable levels of JNK signaling activation (Dillard et al., 2024). While Toll signaling-induced overgrowth in cooperation with Ras^V12^ is independent of the JNK pathway (Brutscher et al., 2024), the strong activation of JNK signaling downstream of Toll is suggested to play an important role during tumor progression (discussed in Section 3.2) (Dillard et al., 2024).

### 3.2 Tumor progression

The ability of systemic innate immune activation to protect epithelia from oncogenic expansion of abnormally developed cells, as in response to loss of apicobasal polarity alone (Parisi et al., 2014) (discussed in Section 2.3), is impaired in the context of malignant tumor cells. Malignant tumor cells are, for example, induced by concomitant activation of oncogenic Ras and loss of apicobasal polarity, e.g., *Ras* ^*V12*^,*scrib*.

In *Ras* ^*V12*^,*scrib*-transformed cells, as part of the systemic immune response, TAH-derived Egr no longer induces tumor cell death as in response to polarity-deficient epithelia (Cordero et al., 2010; Parvy et al., 2019), but promotes malignancy (Cordero et al., 2010). It is therefore tempting to speculate that systemic Toll activation in adipocytes of larvae bearing *Ras* ^*V12*^, *scrib* tumors in the eye/antennal imaginal epithelium (Parisi et al., 2014) no longer promotes tumor cell death, but also contributes to tumor progression.

While the effect of systemic Toll pathway activation on malignant tumors remains elusive, recent research addressed the tumor-autonomous function of Toll signaling in the progression of malignant tumors induced in the eye/antennal imaginal epithelium. There is cumulative evidence that the Toll pathway promotes tumor progression through activation of JNK signaling (Figure 1G) (Dillard et al., 2024; Mishra-Gorur et al., 2019; Ding et al., 2022), a pathway well established to trigger tumor cell invasion through activation of matrix metalloproteinase gene *mmp1* (Uhlirova and Bohmann, 2006). The EMT factors, Snail and Twist have been suggested to mediate the activation of the JNK pathway downstream of Toll signaling in *Ras* ^*V12*^,*scrib* cells (Dillard et al., 2024). Another study shows that Tl-7-dependent endocytosis of Egr facilitates JNK pathway activation to promote the invasion of *Ras* ^*V12*^, *lgl* cells (Ding et al., 2022). Interestingly, in this model, Toll signaling is suggested to accelerate tumor growth through positive regulation of Epidermal growth factor receptor (EGFR) levels, although the mechanism behind this is still unclear (Ding et al., 2022).

*Ras* ^*V12*^,*scrib* tumor cells, derived from the eye/antennal epithelium, frequently invade distal regions of the brain, i.e., the ventral nerve cord and occasionally even target tissues that are located further away from the tumor origin (Pagliarini and Xu, 2003; Igaki et al., 2006). Spz was found to be expressed in target tissues, such as, for example, in the larval salivary gland and adipose tissue, and act as a chemoattractant, guiding tumor cell invasion through interaction with Tl-6 in *Ras* ^*V12*^,*lgl* cells (Mishra-Gorur et al., 2019).

In contrast to the eye/antennal imaginal epithelium, in the wing imaginal epithelium Toll signaling was reported to inhibit tumor growth and progression (Snigdha et al., 2021). The Toll pathway target and negative regulator Cactus accumulates in malignant wing imaginal tumor cells after concomitant activation of oncogenic Ras and impaired apicobasal polarity (*scrib* ^*RNAi*^) (Snigdha et al., 2021). Cactus was suggested to be regulated by Yki and to promote JNK-dependent tumor growth and progression (Snigdha et al., 2021). It remains to be seen whether the upregulated Cactus levels in this context indeed inhibit Toll signaling, but still trigger JNK activation and promote tumor progression.

### 3.3 Tissue overgrowth triggered by “undead” cells

As discussed in the previous chapters, the Toll pathway regulates cell death in various contexts through the upregulation of pro-apoptotic genes. The subsequent apoptotic cascade involves the activation of the initiator caspase Dronc, which promotes the activation of the effector caspases DrICE and Dcp-1 to induce cell death.

If the apoptotic cascade cannot be completed because the effector caspase activity is inhibited by expression of the baculoviral anti-apoptosis protein P35, then the cells remain trapped in an “undead”-like state (Clem et al., 1991; Hay et al., 1994). In these “undead” cells, the activity of Dronc triggers apoptosis-induced compensatory over-proliferation (AiP) of surrounding WT cells. AiP involves the recruitment of circulating hemocytes and activation of JNK signaling in “undead” cells (recently reviewed in (Diwanji and Bergmann, 2019; Fogarty and Bergmann, 2017)). Intriguingly, AiP is suggested to contribute to various forms of cancer (Ryoo and Bergmann, 2012).

A recent study suggests Toll signaling may regulate AiP (Figure 1H) (Shields et al., 2022). Tl-9 was upregulated downstream of JNK in “undead” (*hid-, p35* ^*high*^) cells and was required for AiP in surrounding WT cells. Interaction of Tl-9 with Tl-1 induces Toll signaling and increases the expression of the pro-apoptotic genes *hid* and *rpr*. This in turn establishes a feedback amplification loop promoting AiP through the generation of ROS, recruitment of circulating hemocytes and activation of JNK signaling in “undead” cells. Similarly, Tl-9, has also been reported to induce pro-apoptotic gene expression in the context of *RpL14/+-*induced cell competition (Meyer et al., 2014) suggesting similarities between the local innate immune responses to unfit and undead cells. The heterologous interaction of TRRs, as seen here with Tl-9 and Tl-1, may provide specificity and complexity to the signaling outcome downstream of canonical Tl-1/NF-κB signaling in different contexts and presents an interesting avenue for future research.

## 4 Conclusion and perspectives

The Toll pathway plays important but opposing roles during developmental tissue homeostasis and cancer. Different cellular contexts and hence the integration with different signaling pathways, appears to determine the overall response to Toll signaling activity. To preserve tissue homeostasis or protect from oncogenic transformation, Toll signaling eliminates abnormally developed cells through the induction of apoptosis. In contrast, in the context of cancer, Toll signaling promotes tumor growth and progression, thus serving as an oncogenic factor. Tumor cells evade the Toll-related homeostatic, i.e., growth-inhibiting functions and hijack the Toll-induced target effectors to their own advantage. We discussed different models underlying Toll-dependent tissue overgrowth: a) The Toll pathway can indirectly induce tumorous overgrowth through the selective killing of WT cells adjacent to tumor cells; b) In contrast, it can also promote overgrowth by directly increasing the proliferation of tumor cells. Depending on the genetic context, different signaling pathways have been described to mediate the Toll-dependent proliferative response; c) Toll signaling promotes overgrowth through fueling a feedback amplification loop in the context of apoptosis-induced proliferation.

It will be interesting to further explore how Toll signaling can both promote and inhibit growth. The interaction of multiple TRRs, as proposed in (Shields et al., 2022) or the different TRR-Spz interactions (Table 1) may help define signaling specificity in different cellular contexts. Similarly, in mammals, different TLRs have different functions on cancer cells and can promote or suppress tumor growth, such as by differentially regulating cancer cell apoptosis and proliferation (Yu et al., 2013).

In addition, the multifaceted responses to Toll signaling activity during tissue homeostasis and in different cancer contexts may also depend on the nuclear concentration of Dorsal, as suggested in (Dillard et al., 2024). Similarly, this relationship defines the dorsoventral patterning during *Drosophila* embryogenesis (Roth et al., 1989).

The *Drosophila* larva provides a powerful *in vivo* platform to not only study the effects of local, but also systemic Toll signaling activity on tissue homeostasis and cancer. Therefore, it will be interesting to explore whether and how local and systemic Toll signaling activity in response to developmental or cancerous cues interact to mediate signaling specificity.

Given the high degree of conservation with mammalian TLR/NF-κB signaling, the insights into the functions of Toll signaling in tissue homeostasis and cancer from *Drosophila* research hold great potential. They may help drive future progress in deciphering the complexity of TLR/NF-κB signaling in different cancer contexts, which is critical for the development of therapeutics targeting TLR/NF-κB signaling.
